# The Emerging Role of the RNA-Binding Protein SFPQ in Neuronal Function and Neurodegeneration

**DOI:** 10.3390/ijms21197151

**Published:** 2020-09-28

**Authors:** Yee Wa Lim, Dylan James, Jie Huang, Mihwa Lee

**Affiliations:** Department of Biochemistry and Genetics, La Trobe Institute for Molecular Science, La Trobe University, Melbourne, Victoria 3086, Australia; yeewalim@gmail.com (Y.W.L.); dpjames24@gmail.com (D.J.); huangjie_09241@163.com (J.H.)

**Keywords:** SFPQ, DBHS protein family, *Drosophila* behavior human splicing, RNA-binding protein, nuclear protein, neurodegenerative disease, cytoplasmic aggregation, stress granules

## Abstract

RNA-binding proteins (RBPs) are a class of proteins known for their diverse roles in RNA biogenesis, from regulating transcriptional processes in the nucleus to facilitating translation in the cytoplasm. With higher demand for RNA metabolism in the nervous system, RBP misregulation has been linked to a wide range of neurological and neurodegenerative diseases. One of the emerging RBPs implicated in neuronal function and neurodegeneration is splicing factor proline- and glutamine-rich (SFPQ). SFPQ is a ubiquitous and abundant RBP that plays multiple regulatory roles in the nucleus such as paraspeckle formation, DNA damage repair, and various transcriptional regulation processes. An increasing number of studies have demonstrated the nuclear and also cytoplasmic roles of SFPQ in neurons, particularly in post-transcriptional regulation and RNA granule formation. Not surprisingly, the misregulation of SFPQ has been linked to pathological features shown by other neurodegenerative disease-associated RBPs such as aberrant RNA splicing, cytoplasmic mislocalization, and aggregation. In this review, we discuss recent findings on the roles of SFPQ with a particular focus on those in neuronal development and homeostasis as well as its implications in neurodegenerative diseases.

## 1. Introduction

Near identical DNA is present in every cell of an individual organism, yet it is the presence of protein factors and regulatory RNAs in transcriptional and post-transcriptional processing that are vital for producing the temporal and spatial cellular diversity observed [1]. There are a plethora of transcription factors at play and proteins that interact with and modulate the activity of these factors thus play important roles in the development and maintenance of every cell. To regulate gene expression, factors with the ability to interact with both nucleic acids and proteins are essential for the integration of numerous cellular pathways. A vast volume of intricate networks and the regulation of these interactions are achieved by RNA-binding proteins (RBPs) [2]. Unsurprisingly, the misregulation of RBPs causes changes in gene expression, and in some cases results in the mislocalization of these RBPs as observed in neurological disorders [3,4].

Splicing factor proline- and glutamine-rich (SFPQ) (also known as PTB-associated splicing factor (PSF)) is one such exemplary RBP that has gained recent momentum in research with its emerging roles and implications in neurodegenerative diseases. SFPQ is a ubiquitous and abundant nuclear RBP that belongs to the *Drosophila* behavior/human splicing (DBHS) protein family, a highly conserved group of nuclear proteins exclusively found in the animal kingdom [5]. The DBHS family proteins have an ever-growing list of both protein and nucleic acid binding partners and consequently are implicated in many aspects of nuclear functions including RNA biogenesis and transport, subnuclear body (paraspeckle) formation, as well as DNA damage repair. A more comprehensive review of the DBHS family proteins as a whole can be found elsewhere [5]. In this review, we briefly describe the structural insights and previously reported functions of SFPQ. We then discuss recent findings on the emerging role of SFPQ in neuronal function and homeostasis, in addition to its implications in neurodegenerative diseases.

## 2. The RNA-Binding Protein SFPQ

The DBHS family proteins are RNA-binding proteins that have numerous vital functions in gene regulation throughout both transcriptional and post-transcriptional processes, by interacting with various interaction partners including DNA, RNA, and proteins [5]. Along with SFPQ, two more DBHS family proteins exist in higher vertebrates: Non-POU domain-containing octamer-binding protein (NONO), and paraspeckle protein component 1 (PSPC1), whereas lower vertebrates or invertebrates typically encode a single DBHS protein.

### 2.1. Structure of SFPQ

**Figure 1:**
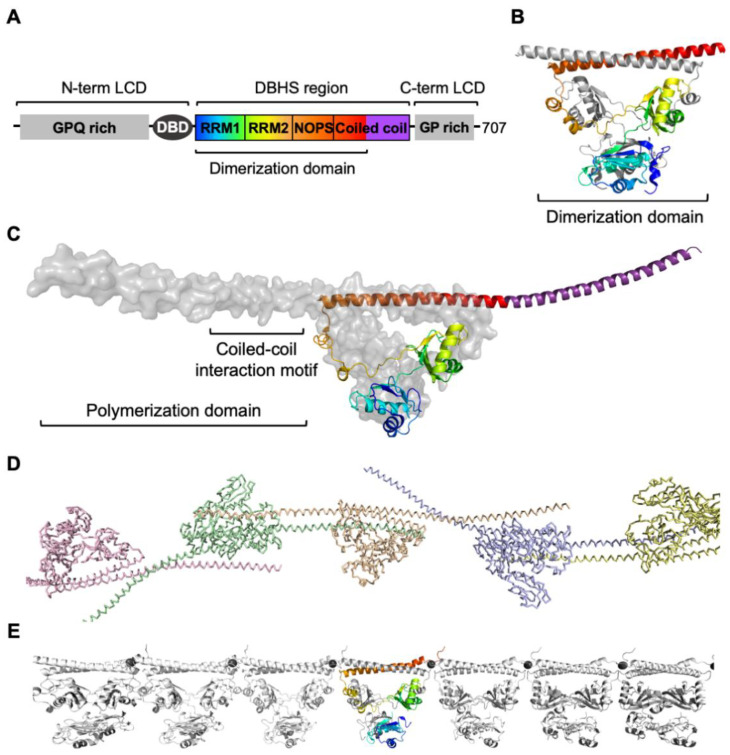
Crystal structures of human splicing factor proline- and glutamine-rich (SFPQ). (**A**) Schematic domain organization of human SFPQ (major isoform, SFPQ-A). The conserved *Drosophila* behavior/human splicing (DBHS) region consists of RNA-recognition motifs (RRM1 and RRM2), NonA/paraspeckles (NOPS) domain, and the coiled-coil domain, flanked by the N- and C-terminal low-complexity domains (LCDs). SFPQ has a DNA-binding domain (DBD) of which boundary remains to be defined. (**B**) Crystal structure of the dimerization domain of human SFPQ (PDB ID: 6NCQ) [9] with one monomer in the same color scheme in (**A**) and the other in grey. (**C**) Crystal structure of the full DBHS-region of the human SFPQ homodimer (PDB ID: 4WIJ) [7] with one monomer in the same color scheme in (**A**) and the other in grey. (**D**) Infinite polymer of SFPQ (PDB ID: 4WIJ) [7]. Polymerization of SFPQ mediated by the coiled-coil interaction motif is critical for the cellular function of SFPQ. (**E**) Zinc-mediated infinite polymerization of SFPQ (PDB ID: 6OWJ) that may represent a pathological form of polymerization under high zinc condition [10] (further discussion in Section 4.1). Zn(II) atoms are depicted as black spheres.

A key feature that defines DBHS proteins is the evolutionarily conserved DBHS region, which encompasses ~300 amino acids. This region is composed of two tandem RNA-recognition motifs (RRM1 and RRM2), a NonA/paraspeckles (NOPS) domain, and a C-terminal coiled-coil domain (Figure 1A). SFPQ also harbors the N- and C-terminal low-complexity domains flanking the DBHS region. Although the precise boundary of the DNA-binding domain is yet to be defined, it is known that the sequence N-terminal to RRM1 is required for DNA-binding activity [6,7]. Two isoforms of SFPQ have been reported with the major isoform A (SFPQ-A) containing 707 amino acids and a shorter spliced isoform (SFPQ-F) containing 669 amino acids. The shorter spliced isoform shares the same sequence to amino acid 662 of SFPQ-A, but diverges with seven additional amino acids, lacking the nuclear localization signal present at the C-terminal end of the major isoform [8].

Crystal structures of the DBHS domain of SFPQ have been previously reported [7,9,10]. These structures show the dimeric nature of SFPQ with extensive intermolecular interactions present within the dimer, which are primarily governed by hydrophobic interaction (Figure 1B). The second RRM (RRM2), NOPS, and the coiled-coil domain provide the major dimerization interface. The NOPS domain was defined based on the PFAM sequence alignment among the DBHS proteins and is unique to the family; it corresponds to a linker region connecting the RRM2 and coiled-coil domains. The NOPS domain of one monomer makes extensive interactions with RRM2 of the second monomer, followed by antiparallel coiled-coil interactions (Figure 1B).

Owing to the unusual antiparallel configuration of the coiled-coil domains, the crystal structure of the full-length DBHS region reveals a strikingly extended structure of over 260 Å [7] (Figure 1C). The coiled-coil domain of SFPQ extends out from the globular dimer core of the protein and provides the platform for polymerization with the corresponding coiled-coil of neighboring dimers (Figure 1D). Complementing the crystal structure, small-angle X-ray scattering data, and cell-biology approaches showed that the polymerization of SFPQ is dynamic, reversible, and dependent on protein concentration, as well as being critical for protein function [7]. Disruption of polymerization by truncation of the coiled-coil domain or mutation of the coiled-coil interaction motif, results in attenuated DNA-binding capacity and reduced transcriptional activation, while also reducing the formation of paraspeckles [7]. Therefore, impaired polymerization of SFPQ may present potential molecular mechanisms for the misregulation of SFPQ [10,11] (further discussion in Section 4.1).

DBHS proteins including SFPQ function as obligate dimers; this has been demonstrated through previous structural characterizations using X-ray crystallography and small-angle X-ray scattering [7,9,10,12,13,14]. Dimerization of the DBHS proteins is not only critical to their structural integrity but also to their functions such that dimerization incompetent DBHS proteins generated by mutation of key hydrophobic residues failed to form paraspeckles [12]. Within the DBHS region, the three human DBHS proteins share more than 70% sequence identity. It is noteworthy that the DBHS proteins also form heterodimers [12,15], allowing six possible dimer combinations in higher vertebrates (three heterodimers and three homodimers). In fact, heterodimerization is preferred over homodimerization [13]. It was proposed that the differences in dimerization affinity may represent a potential mechanism by which PSPC1 at a lower relative cellular abundance can outcompete NONO to heterodimerize with SFPQ [13]. Consistent with these observations of DBHS proteins, the NOPS domain of SFPQ is more flexible than other domains, which may indicate the need for flexibility in the exchange of dimer partners at different cellular contexts and developmental stages [9]. However, this also imposes difficulties in deciphering individual contributions to certain functions. Thus, some of the descriptions in the next section may be the consequences of the heterodimers (SFPQ/NONO and/or SFPQ/PSPC1) rather than those of the SFPQ homodimer.

### 2.2. Reported Nuclear Function of SFPQ

SFPQ has been labeled as a “multifunctional” protein due to its ability to interact with a large pool of both nucleic acid and protein interaction partners [5,16,17]. In this section, some of the previously reported nuclear functions of SFPQ are presented in the contexts of paraspeckle formation, alternative splicing, transcriptional regulation, DNA damage repair, and maintenance of genome stability.

#### 2.2.1. Paraspeckle Formation

**Figure 2:**
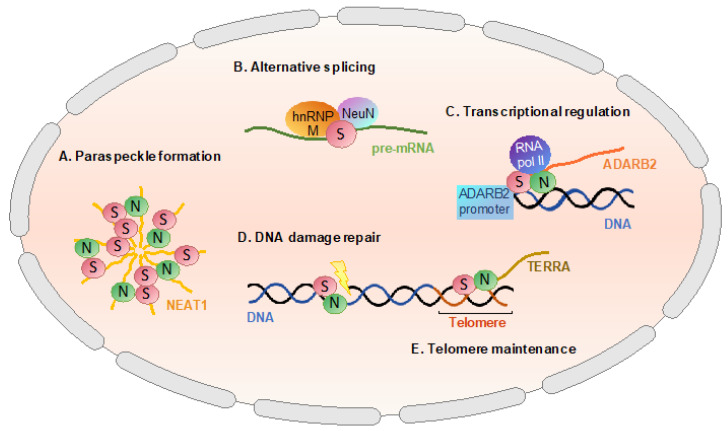
The nuclear function of SFPQ. SFPQ is critical for the structural integrity of paraspeckles (**A**). SFPQ interacts with other splicing factors such as hnRNP M and NeuN to regulate alternative splicing of pre-mRNA (**B**). SFPQ acts as an important transcriptional coactivator by interacting with RNA Polymerase II (**C**). SFPQ is also involved in DNA damage repair—both in homologous repair and nonhomologous end-joining pathways (**D**). SFPQ facilitates telomere maintenance, interacting with a telomere repeat-containing long noncoding RNA, TERRA (**E**). SFPQ is depicted as red circles labeled with “S” while its dimerization partner NONO as green circles with “N”.

Paraspeckles are mammalian-specific membraneless subnuclear bodies composed of RNAs and proteins [18]. Along with the architectural long noncoding RNA, nuclear paraspeckle assembly transcript 1 (NEAT1), SFPQ is critical for the structural integrity of paraspeckles (Figure 2A). The knockdown of SFPQ leads to the loss of paraspeckles in HeLa cells, indicating the crucial role of SFPQ in the formation of these subnuclear bodies [19,20]. Accumulating evidence has demonstrated that a dynamic liquid demixing process, known as liquid–liquid phase separation, underlies the formation of membraneless organelles including paraspeckles [21]. NEAT1 serves as a scaffold for protein binding, seeding paraspeckle formation; SFPQ and its paralogue NONO bind NEAT1 to form a minimal ribonucleoprotein particle, which triggers polymerization of SFPQ (and presumably NONO). It is hypothesized that the low-complexity domains of SFPQ interact with additional proteins found in paraspeckles most of which also harbor low-complexity domains, inducing liquid–liquid separation to form paraspeckles [22]. While the precise functions of paraspeckles and the underlying mechanisms remain to be elucidated, it is generally accepted that they are involved in gene regulation in response to certain stress conditions, such as proteasomal inhibition and viral infection [23].

#### 2.2.2. Alternative Splicing

The initial characterization of SFPQ in the early 1990s classified the protein as a splicing factor, owing to its association with polypyrimidine tract-binding protein (PTB) and its requirement in the early spliceosome formation [8]. Several other splicing factors have been identified as interaction partners of SFPQ since the initial characterization, giving additional validity to the role of SFPQ in alternative splicing. Heterogeneous nuclear ribonucleoprotein (hnRNP) M is one of these proteins and it has been shown that the overexpression of SFPQ promotes increased exon inclusion in a splicing minigene reporter, presumably through interaction with hnRNP M [24]. The hnRNP family is heavily involved in the splicing processes in the nucleus, predominantly through association with pre-mRNA substrates by family members [25]. NeuN (also known as Rbfox3 and Fox3) is another example of alternative splicing factors shown to interact with SFPQ. Evidence suggests that SFPQ is a coactivator of NeuN, assisting in the recruitment of NeuN to a motif that enhances the inclusion of exon N30 of nonmuscle myosin heavy chain (NMHC) II-B in the mouse central nervous system [26]. Additionally, hnRNP M and NeuN have been shown to act as part of the same splicing regulation complex [27]. Both proteins being known associates of SFPQ further supports the role of SFPQ in alternative splicing (Figure 2B).

In contrast, the association of SFPQ with the exonic splicing silencer 1 (ESS1)-bound hnRNP L complex was shown to promote exon skipping of the CD45 gene. ESS1 is a motif found on exon 4 of the CD45 gene; hnRNP L is typically bound to this site in resting T cells preventing the splicing of exons 4–6 [28]. It has been proposed that T cell activation allows the recruitment of SFPQ to the ESS1 element, negating the interference of hnRNP L and generating a CD45 variant that is vital for activated T cell behavior [29,30].

#### 2.2.3. Transcriptional Regulation

In the context of transcriptional regulation, SFPQ has been shown to act as a corepressor as well as a coactivator, depending on the cellular context. The most prominent illustration of SFPQ behaving as a coactivator is the way the protein is entwined in the transcriptional machinery of the cell. SFPQ/NONO has been heavily implicated in the initiation and elongation stages of transcription via direct interaction with the carboxyl-terminal domain of RNA Polymerase II [31,32]. This association is thought to allow SFPQ/NONO to act as a bridge between RNA Polymerase II and promoter-bound transcriptional activators, initiating transcription. Thus, SFPQ/NONO, in helping to build the skeleton of the nascent transcriptional machinery, essentially acts as an important coactivator in many gene expression scenarios [33]. For example, SFPQ was observed to increase the expression of the adenosine deaminase B2 (ADARB2) gene through interaction with the ADARB2 promoter [34] (Figure 2C).

The contrasting role of SFPQ as a corepressor has been demonstrated when interacting with members from the nuclear hormone receptor (NHR) family. The activation and repression of downstream genes by NHRs follow a canonical ligand-dependent regulation system, where activation or repression is conditional on the binding of a specific ligand to the receptor [35]. SFPQ has been reported to repress gene expression through interaction with the DNA-binding domain of apo-NHRs including progesterone receptor (PR), androgen receptor (AR), thyroid hormone receptors (TR), retinoid X receptor (RXR), and peroxisome proliferator-activated receptor (PPAR) [36,37,38]. This binding then initiates the recruitment of corepressor Sin3A, which provides a platform for the assembly of histone deacetylases (HDACs), leading to chromatin condensation and transcriptional repression [38]. Interestingly, the major long isoform SFPQ-A, but not the shorter spliced isoform SFPQ-F, was shown to repress AR transcriptional activity on AR [37], suggesting that the lack of the nuclear localization signal in SFPQ-F may attribute to the functional difference of the two isoforms. Another example utilizing a similar mechanism is the interaction of SFPQ with signal transducer and activator of transcription 6 (STAT6), where SFPQ bridges the interaction between STAT6 and Sin3A, again recruiting HDACs to the complex for transcriptional repression [39].

#### 2.2.4. DNA Damage Repair and Maintenance of Genome Stability

DNA damage repair is a critical process that maintains genome stability and integrity, which is essential for cellular homeostasis and the prevention of neoplasia, the formation of abnormal tissue growth. The SFPQ/NONO heterodimer has been shown to interact with many of the proteins involved in various DNA damage repair pathways. Consequently, SFPQ has been implicated in both of the major DNA damage repair processes in eukaryotic cells, namely the nonhomologous end-joining (NHEJ) and homologous recombination (HR) pathways.

A biochemical screen of DNA end-joining proteins identified SFPQ/NONO and highlighted its involvement with the KU70/KU80 heterodimer in binding substrate DNA to form a preligation complex [40]. Additional investigation revealed that the SFPQ/NONO dimer dramatically increases DNA end-joining through direct interaction with DNA substrates in vitro [40]. In addition, the SFPQ/NONO heterodimer has been shown to interact with IGFBP3, which forms a complex with EGFR and DNA-PKcs in triple-negative breast cancer cells, contributing to the IGFBP3 dependent NHEJ [41].

Furthermore, upon induction of DNA damage by laser microbeam, the rapid recruitment of the SFPQ/NONO heterodimer was observed, indicating their involvement in the early stages of the DNA damage response [6,42] (Figure 2D). This suggestion was further confirmed by the observed interaction between SFPQ/NONO and Matrin 3 (MATR3) [42]. MATR3 is a target of the nuclear kinase Ataxia Telangiectasia Mutated (ATM), which is one of the first responders on the scene for the double-strand DNA break repair process. Evidence indicates that MATR3 may coordinate both the NHEJ and HR pathways for DNA repair, with reports of interactions with Ku70/Ku80 and DNA ligase IV from the NHEJ process and regulation of RAD51 from the HR pathway [42,43]. A recent study has also demonstrated that SFPQ/NONO substitutes XLF, an accessory factor of DNA ligase IV, suggesting that SFPQ/NONO provides an alternative mechanism to facilitate DNA substrate pairing during the ligation phase of the reaction in NHEJ [44]. In addition, SFPQ has been shown to interact directly with RAD51, an essential recombinase in HR, modulating the homologous-pairing and strand-exchange activities of RAD51 [45].

More recently, further involvement of SFPQ in the maintenance of genome stability has been demonstrated in relation to telomere maintenance, where the SFPQ/NONO heterodimer has been identified as a novel regulator at telomeres [46] (Figure 2E). It was shown that loss of SFPQ/NONO results in a significant increase in telomere recombination events and rapid alterations in telomere length in both telomerase-positive and negative cells. Mechanistically SFPQ/NONO binds a telomere repeat-containing long noncoding RNA, TERRA, to suppress the RNA–DNA hybrid formation, DNA replication defects, homologous recombination, and DNA damage at telomeres [46].

## 3. SFPQ in Neuronal Development and Maintenance of Neuronal Function

Gene regulation plays a fundamental role during the complex process of neuronal development in supporting the development and maintaining homeostasis. Many RNA-binding proteins are directly or indirectly involved in mediating gene regulations during transcriptional, post-transcriptional, and translational processes [47]. The diverse cellular roles of SFPQ described above also directly affect cell homeostasis during neuronal development, differentiation, proliferation, and maintenance. It appears that the versatility of SFPQ being able to dimerize with its DBHS paralogues as well as interacting with other proteins, RNA and DNA contributes to its multiple roles in the nucleus, cell body, and axon of various types of neuron. This section describes the recent findings on the role of SFPQ in the context of neuronal development and homeostasis.

### 3.1. Transcriptional and Post-Transcriptional Regulation to Develop and Maintain Neuronal Function

**Figure 3:**
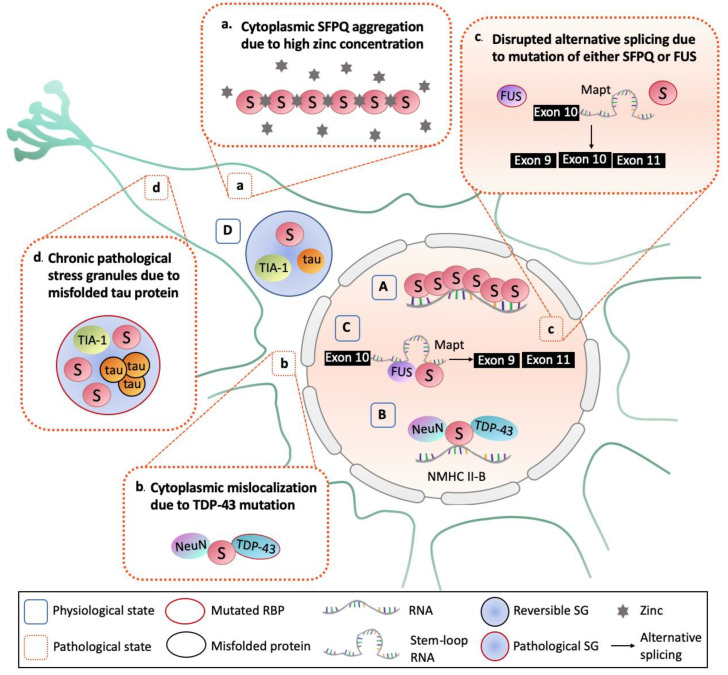
Schematic comparison of the neuronal functions of SFPQ in physiological conditions (**A**–**D**) with pathological features of SFPQ in the disease states (**a**–**d**). (**A**) SFPQ is critical for transcriptional elongation of long genes in the developing brain while the cytoplasmic aggregation and mislocalization of SFPQ are observed in many neurodegenerative diseases including, amyotrophic lateral sclerosis (ALS), frontotemporal lobar degeneration (FTLD), and Alzheimer’s disease (AD). One possible mechanism of cytoplasmic aggregation and mislocalization of SFPQ caused by high zinc concentration in the cytosol is shown (**a**). (**B**) The alternative splicing of the NMHC II-B mRNA by NeuN and SFPQ in supporting neuronal development is influenced by TDP-43 mutation causing cytoplasmic mislocalization in the disease state (**b**). (**C**) Alternative splicing of the *Mapt* gene to form different isoforms of tau, is mediated by SFPQ and FUS; altered splicing is caused by mutation of either of these two RBPs (**c**). (**D**) SFPQ, TIA-1, and tau form reversible stress granules (SGs) under stress, which become persistent and pathological SGs in the disease state (**d**). SFPQ is depicted as red circles labeled with “S”

The two main stages in the transcriptional process—initiation and elongation—are fundamental in determining the gene expression that is required for the differentiation, growth, and homeostasis of cells and tissues [48]. In the development of the brain, much longer pre-mRNA genes with long introns are expressed [49]. It has been shown that SFPQ regulates the transcriptional elongation of extra-long genes over 100 kb in the developing mouse brain, by cotranscriptionally binding to target pre-mRNAs with extra-long introns [50,51]. It was hypothesized that SFPQ polymers bind to and stabilize the long pre-mRNA in neurons to facilitate transcriptional elongation by recruiting cyclin-dependent kinase 9 (CDK9) and activating RNA polymerase II [50] (Figure 3A). In addition, loss of SFPQ specifically causes impaired transcriptional elongation, downregulating long genes in the developing mouse brain and leading to neuronal apoptosis [50,51]. Many of these downregulated long genes are associated with neurodegenerative and psychiatric diseases, and are essential for axon guidance, neuronal migration, and synapse formation, indicating the essential neuroprotective role of SFPQ in gene expression during neuronal development and survival.

SFPQ is also known to regulate alternative splicing of pre-mRNA after the initial stage to provide proteome diversity in higher vertebrates. As described in Section 2.2.2, the interaction of SFPQ with NeuN (also known as Rbfox3 and Fox-3) activates neuronal cell-specific alternative splicing. It was shown that SFPQ enhances the NeuN-dependent inclusion of an exon (N30) of nonmuscle myosin heavy chain (NMHC II–B) mRNA to encode the NMHCII-B protein required for neuronal function via regulation of MNDA receptor trafficking (Figure 3B) [26]. Another independent study also demonstrated the interaction between SFPQ and NeuN in the murine cell line, N2A cells, and pig brain [52], reporting coimmunoprecipitation of SFPQ, NeuN, and TAR DNA-binding protein 43 (TDP-43); this interaction was further enhanced in transgenic pigs expressing mutated TDP-43 (M337V) and resulted in cytoplasmic mislocalization of SFPQ and NeuN along with the mutated TDP-43, causing abnormal NMHC 11-B splicing (further discussion in Section 4.2).

SFPQ has also been shown to form an intranuclear complex with another RBP, Fused in sarcoma (FUS), and regulate alternative splicing of the *Mapt* gene, which encodes the tau protein (Figure 3C) [53]. Tau is a microtubule-associated protein in mature neurons and the main component of the neurofibrillary tangles found in many neurodegenerative diseases [54]. Loss of either SFPQ or FUS has been shown to disrupt the tau isoform equilibrium, leading to neuronal loss (further discussion in Section 4.2) [53].

From a broader perspective, an earlier study of neurogenesis in zebrafish found that SFPQ is important for the survival and differentiation of neuronal cells [55]. Zebrafish embryos expressing truncated SFPQ mutants showed abnormal brain development and died by 4 days postfertilization. Immunohistochemistry using markers for neuronal differentiation showed that all reticulospinal neurons in the hindbrain except Mauthner neurons were absent in the zebrafish *sfpq* mutant embryos while no loss of motoneurons was observed in the brain or in the spinal cord, indicating that SFPQ has specific roles in the differentiation of certain neuronal cell types [55].

### 3.2. The Role of the Cytoplasmic Pool of SFPQ in Axon Growth and Survival

Although SFPQ is found primarily in the nucleus, a small subset of the total cellular pool of SFPQ has been observed in the cytoplasm of motor axons and shown to drive axon maturation and connectivity [11]. Using a genetic screen for abnormal neural development in zebrafish embryos, a null mutation in *sfpq* was discovered which was rescued by microinjecting human *sfpq* mRNA [11]. The authors also showed that the cytoplasmic pool of SFPQ is critical for the motor axon development by rescuing the neuronal defect in the mutant zebrafish with human SFPQ lacking the nuclear localization signal. This study established that SFPQ has a conserved function in the development of motor axon in zebrafish and humans—a non-nuclear pool of SFPQ regulates axonal transcripts required for normal motor neuron development.

In another study, colocalization of SFPQ/NONO with another abundant RBP, HERMES (also known as RBPMS), and the stress granule component G3BP1 were observed when neuronal differentiation was induced in retinal ganglion cells, resulting in the formation of cytoplasmic neuronal granules [56]. Colocalization of kinesin (KIF5), an RNA transport granule marker, with SFPQ/NONO, HERMES, and G3BP1 suggested that these cytoplasmic neuronal granules are RNA transport granules, which are important for mRNA transport to dendrites for local protein synthesis in neurons. This is consistent with the previous finding that SFPQ and NONO are associated with RNA transport granules [57]. Knockdown of SFPQ has been shown to cause suppression of RNA transport to dendrites [57]. These data indicate that the cytoplasmic pool of SFPQ plays a role in the maintenance of neurons.

Similarly, SFPQ was also found to interact and guide multiple axonal mRNAs such as *laminb2* and *bclw* in dorsal root ganglion sensory neurons to support axon survival [58]. It has been shown that SFPQ assists the transportation of *laminb2* and *bclw* mRNA from the nucleus to the axon by RNA transport granules. LaminB2 and Bclw proteins are then translated for the use of maintaining axonal survival and functions. When SFPQ was knocked down, the protein expression level of LaminB2 and Bclw in the axon decreased, leading to axon degeneration. This indicates the function of SFPQ in regulating the level of *laminb2* and *bclw* mRNA in the axon for translation and in turn, supporting axonal survival [58].

More recently, it has been demonstrated that a complex containing RNA and SFPQ directly interacts with a tetrameric kinesin complex containing the motor, KIF5A, and the adaptor, KLC1 [59], further evidencing the SFPQ-dependent mRNA transport in neurons. Defects in kinesin-driven transport of SFPQ led to axon degeneration in the dorsal root ganglion sensory neurons, indicating that the interaction of SFPQ with the kinesin motor complex sustains axon survival by transporting relevant mRNA towards the distal end of the dorsal root ganglion sensory neurons for translation. Interestingly, the authors found that the interaction of the kinesin motor complex and SFPQ is RNA-dependent, suggesting that KIF5A/KLC1 bind and transport SFPQ when it is part of large RNP transport granules [59].

Collectively, these data strongly indicate that SFPQ plays important roles in the nucleus and axon of neurons, and that the balance in the nucleocytoplasmic distribution of SFPQ is critical for the development and maintenance of neuronal integrity and function.

## 4. Implications of SFPQ in Neurodegenerative Diseases

Not surprisingly, due to the emerging physiological roles of SFPQ in neuronal development and homeostasis as described above, an increasing number of recent literature has demonstrated the implications of SFPQ in neurodegenerative diseases such as amyotrophic lateral sclerosis (ALS) frontotemporal lobar degeneration (FTLD), and Alzheimer’s disease (AD) [60,61,62,63,64]. Neurodegenerative diseases cause the progressive loss of physical and cognitive function, which ultimately leads to death [65,66]. Pathological protein aggregation, manifested as amyloid plaques and neurofibrillary tangles, is a hallmark of a number of neurodegenerative diseases [67,68]. In this section, we will focus on the emerging neuropathological features of SFPQ including misregulated alternative splicing processes, imbalance in nucleocytoplasmic distribution, the formation of cytoplasmic aggregation, and other cellular complications.

### 4.1. Nuclear Depletion and Cytoplasmic Aggregation of SFPQ

The cytoplasmic mislocalization and aggregation of RBPs have been emerging as new hallmarks of neurodegenerative diseases [3,4,69]. The most notable examples are TDP-43, and FUS, which have been implicated in the onset and progression of ALS and FTLD [70]. These RBPs are predominantly localized to the nucleus under normal conditions, however, imbalanced nucleocytoplasmic distribution is observed in the disease state where they form cytoplasmic aggregates. An increasing number of recent literature has also demonstrated the nuclear depletion and cytoplasmic aggregation of SFPQ in the neurodegenerative diseases including ALS, FTLD, and AD [60,61,62,63,64].

The molecular mechanisms by which the nuclear pool of SFPQ is mislocalized to the cytoplasm and forms aggregates remain to be defined. However, several lines of evidence suggest that aberrant interactions of SFPQ with other misregulated or mutated proteins cause these pathological features. Interestingly, most of these proteins are neurodegenerative disease-implicated proteins such as tau, TDP-43, and FUS. Overexpression of pathological tau mutant (P301L) previously identified in familial cases of FTLD promotes cytoplasmic accumulation and aggregation of SFPQ [61]. The familial ALS mutant TDP-43 (M337V) also resulted in the cytoplasmic mislocalization of SFPQ in the transgenic pig model [52] (Figure 3b). In contrast, the interaction between SFPQ and FUS has been shown to be essential in neuronal homeostasis by maintaining the isoform ratio of tau [53] as such impaired interaction of FUS and SFPQ and the subsequent aberrant ratio of tau was observed in the ALS and FTLD-affected neurons [64]. The colocalization of SFPQ with phosphorylated tau (p-tau) in tau tangles was also observed in the postmortem brains of rapidly progressive AD patients, which led to a conclusion that the mislocalization of SFPQ represents a critical pathway to rapid progression of AD [63] (Figure 3d).

Altered zinc homeostasis has also been proposed as a potential mechanism of the cytoplasmic accumulation and aggregation of SFPQ [10]. Altered metal homeostasis and increased oxidative stress have consistently been proposed as central features of neurodegeneration [71]. In particular, zinc is selectively stored in, and released from the presynaptic vesicles of some neurons, and therefore, its dysregulation can be detrimental to neurons [72]. An unexpected discovery of the zinc-mediated polymerization of SFPQ was demonstrated by the crystal structure (Figure 1E) [10]. Zinc-induced polymerization of SFPQ causes a significant conformational change in the coiled-coil interaction motif, which is most likely to prevent the functional polymerization of SFPQ mediated by the coiled-coil domain, as described in Section 2.1. The subsequent application of high zinc to primary cortical neurons induced the cytoplasmic accumulation and aggregation of SFPQ, leading to the proposal that dysregulation of zinc availability and/or localization in neurons represents a mechanism for the imbalance in the nucleocytoplasmic distribution of SFPQ observed in the neurodegenerative diseases [10] (Figure 3a). This discovery is also in line with the zinc-induced mislocalization and aggregation of TDP-43 [73,74].

Although whether loss of the nuclear pool of SFPQ is a cause or merely an effect of the progression of the diseases is yet to be defined, given the predominant presence of SFPQ in the nucleus and the important nuclear roles of SFPQ in many aspects of RNA biogenesis and the maintenance of genome stability, imbalance in the nucleocytoplasmic distribution of SFPQ is likely to contribute to the onset and/or progression of the diseases by loss-of-function mechanisms (further discussions in Section 4.2 and Section 4.3).

### 4.2. Abnormal RNA Splicing

As previously mentioned in Section 3.1, the interaction of SFPQ and FUS was found to be essential in alternative splicing of the *Mapt* gene, which encodes the tau protein [53]. Hippocampus-specific SFPQ- or FUS-knockdown led to FTLD-like phenotypes in mice including reduced adult neurogenesis, accumulation of p-tau, and hippocampal atrophy with neuronal loss. In a follow-up study, the authors further demonstrated the biological link among SFPQ/FUS, tau isoform alteration, and FTLD-like phenotypes [64], where the examination of postmortem samples from patients of FTLD-spectrum diseases revealed impaired intranuclear colocalization of SFPQ and FUS in neurons of FTLD-spectrum cases including FUS-related ALS/FTLD, TDP-43-related ALS/FTLD (Figure 3c). These data led to the conclusion that impaired interactions between intranuclear FUS and SFPQ and the subsequent aberrant tau isoform ratio constitute a common pathogenesis pathway in FTLD-spectrum diseases.

Abnormal neuronal RNA splicing was further shown in another study with the examination of postmortem samples from individuals with ALS [52]. The neuronal cell-specific alternative splicing regulated by SFPQ/NeuN was impaired in the ALS patient samples, demonstrated by the increased isoforms of the NMHCII-B transcript excluding the N30 exon and the concurrent decreased level of the NMHC II-B protein in the ALS patient cerebellar cortex tissues.

Interestingly, aberrant RNA splicing of the *sfpq* transcript has also been implicated in ALS [62,75]. Abnormal intron retention of the *sfpq* transcript has been observed during the motor neuron differentiation of ALS patient-derived induced-pluripotent stem cells (iPSCs). In addition, SFPQ was shown to bind extensively to its retained intron, which leads to nuclear loss and cytoplasmic accumulation of SFPQ. These data led to the proposal that intron retention of the *sfpq* transcript and nuclear loss of SFPQ are general molecular hallmarks of familial and sporadic ALS [62]. In a follow-up study, the authors demonstrated that FUS also binds to the intron-retained *sfpq* transcript, which promotes the mislocalization of FUS to the cytoplasm [75]. Therefore, aberrant intron retention of *sfpq* transcripts and the interaction of SFPQ and FUS with the retained intron was proposed as an underlying mechanism of mislocalization of SFPQ and FUS in ALS [62,75].

### 4.3. Persistent Pathological Stress Granules

Stress granules are another type of membraneless organelles governed by liquid–liquid phase separation [21]. Stress granules are formed in the cytoplasm in response to cellular stress in order to halt protein production by arresting the translation of mRNAs, and are quickly disassembled when the stress signal is lifted. However, failure in the disassembly of these transient protein–protein or protein–RNA complexes leads to persistent pathological stress granules [76]. A recent study demonstrated the emerging role of SFPQ in aberrant dynamics of stress granules and its implications in the rapid progression of AD [63]. Consistent with the previous finding by Ke et al. [61], this report confirmed a drastic nuclear depletion of SFPQ in the frontal cortex of rapidly progressive AD cases. Importantly, SFPQ was shown to colocalize with p-tau and TIA-1, a stress granule marker RBP, in the cytoplasm of rapid progressive AD-affected postmortem brain lesions, prompting the role of SFPQ in stress granule formation. The authors showed that oxidative stress by arsenite treatment induced cytoplasmic redistribution of SFPQ into stress granules in HeLa cells, which colocalized with TIA-1 as well as p-tau, thereby supporting the role of SFPQ in stress granule formation (Figure 3D). This observation was further enhanced by the expression of pathological mutant tau (P301L) (Figure 3d). Therefore, these data suggest that the cytoplasmic aggregates of SFPQ found in the disease state may constitute persistent pathological stress granules that are no longer reversible and dynamic (Figure 3d).

Many lines of evidence point to cytoplasmic accumulation of SFPQ as an emerging hallmark of neurodegenerative diseases. As mentioned previously, nucleus-to-cytoplasmic redistribution of SFPQ is most likely to cause loss of nuclear function. Whether the cytoplasmic aggregates of SFPQ found in the neurodegenerative diseases confer gain-of-function in the form of cytotoxicity is an intriguing possibility.

## 5. Concluding Remarks

Recent findings on the role of SFPQ and its implications in neurodegenerative diseases are reminiscent of TDP-43 in many ways. Both SFPQ and TDP-43 are RNA- and DNA-binding proteins and play multiple roles in RNA biogenesis and the maintenance of genome stability. A recent finding on the involvement of TDP-43 in DNA damage repair [77] further supports this comparison. They are predominantly found in the nucleus under normal conditions while forming cytoplasmic aggregates in the disease state. Although the two proteins use different domains for the polymerization (the N-terminal domain in TDP-43 [78] versus the C-terminal coiled-coil domain in SFPQ), functional and dynamic polymerization is essential for their function in the nucleus while the cytoplasmic aggregation of both proteins is linked to the neuropathological conditions. Increasing evidence of their involvement in stress response mechanisms by persistent pathological stress granule formation in the neurodegenerative diseases is also intriguing. The low-complexity domains are found in both proteins, which are proposed to confer their ability to drive the liquid–liquid phase transition-mediated granule formation—paraspeckle formation and DNA damage repair foci in the nucleus, and RNA transport granule and stress granule formation in the cytoplasm.

The role of SFPQ, in concert with FUS, in maintaining the isoform ratio of tau, a primary component of neurofibrillary tangles in the neurodegenerative diseases, further interweaves the intricate network of SFPQ with other neurodegenerative disease-linked RBPs. However, the precise molecular mechanisms that coordinate these complicated networks spatially and temporally are yet to be elucidated. Thus, further understanding of the precise mechanisms underpinning the interactions of SFPQ and other RBPs may also reveal the molecular mechanisms underlying the onset and progression of neurodegenerative diseases. Pathological protein aggregation manifested as neurofibrillary “tangles” is a hallmark of a number of neurodegenerative diseases including ALS and AD. SFPQ, as presented in this review, participates in the intricate network with many other known neurodegenerative disease-linked RBPs and tau. Given the complex regulatory roles of SFPQ through interactions with a wide array of interaction partners including DNA, RNA, and proteins, finding ways to “untangle” these interactions and navigate through the intricate regulatory network of SFPQ may present future diagnostic and/or therapeutic avenues for neurogenerative diseases.

## Abbreviations

AD	Alzheimer’s disease
ADARB2	adenosine deaminase B2
ALS	amyotrophic lateral sclerosis
AR	androgen receptor
ATM	ataxia telangiectasis mutated
CDK9	cyclin-dependent kinase 9
DBD	DNA-binding domain
DBHS	Drosophila behavior human splicing
ESS1	exonic splicing silencer 1
FTLD	frontotemporal lobar degeneration
FUS	fused in sarcoma
HDAC	histone deacetylase
hnRNP	heterogeneous nuclear ribonucleoprotein
HR	homologous recombination
iPSC	induced-pluripotent stem cell
LCD	low-complexity domain
MATR3	Matrin 3
NEAT1	nuclear paraspeckle assembly transcript 1
NeuN	neuronal nuclei
NHEJ	nonhomologous end-joining
NHR	nuclear hormone receptor
NMHC	nonmuscle myosin heavy chain
NONO	Non-POU domain-containing octamer-binding protein
NOPS	NonA/paraspeckles
PPAR	peroxisome proliferator-activated receptor
PR	progesterone receptor
PSPC1	paraspeckle protein component 1
PSF	PTB-associated splicing factor
PTB	polypyrimidine tract-binding protein
RBP	RNA-binding protein
RXR	retinoid X receptor
RRM	RNA-recognition motif
SFPQ	splicing factor proline- and glutamine-rich
STAT6	signal transducer and activator of transcription 6
TDP-43	TAR DNA-binding protein 43
TR	thyroid hormone receptor
TERRA	telomere repeat-containing long noncoding RNA

## References

[1] Albert F.W., Kruglyak L. (2015). The role of regulatory variation in complex traits and disease. Nat. Rev. Genet..

[2] Hentze M.W., Castello A., Schwarzl T., Preiss T. (2018). A brave new world of RNA-binding proteins. Nat. Rev. Mol. Cell Biol..

[3] Conlon E.G., Manley J.L. (2017). RNA-binding proteins in neurodegeneration: Mechanisms in aggregate. Genes Dev..

[4] Nussbacher J.K., Tabet R., Yeo G.W., Lagier-Tourenne C. (2019). Disruption of RNA Metabolism in Neurological Diseases and Emerging Therapeutic Interventions. Neuron.

[5] Knott G.J., Bond C.S., Fox A.H. (2016). The DBHS proteins SFPQ, NONO and PSPC1: A multipurpose molecular scaffold. Nucleic Acids Res..

[6] Ha K., Takeda Y., Dynan W.S. (2011). Sequences in PSF/SFPQ mediate radioresistance and recruitment of PSF/SFPQ-containing complexes to DNA damage sites in human cells. DNA Repair (Amst).

[7] Lee M., Sadowska A., Bekere I., Ho D., Gully B.S., Lu Y., Iyer K.S., Trewhella J., Fox A.H., Bond C.S. (2015). The structure of human SFPQ reveals a coiled-coil mediated polymer essential for functional aggregation in gene regulation. Nucleic Acids Res..

[8] Patton J.G., Porro E.B., Galceran J., Tempst P., Nadal-Ginard B. (1993). Cloning and characterization of PSF a novel pre mRNA splicing factor. Genes Dev..

[9] Hewage T.W., Caria S., Lee M. (2019). A new crystal structure and small-angle X-ray scattering analysis of the homodimer of human SFPQ. Acta Crystallogr. Sect. F Struct. Biol. Commun..

[10] Huang J., Ringuet M., Whitten A.E., Caria S., Lim Y.W., Badhan R., Anggono V., Lee M. (2020). Structural basis of the zinc-induced cytoplasmic aggregation of the RNA-binding protein SFPQ. Nucleic Acids Res..

[11] Thomas-Jinu S., Gordon P.M., Fielding T., Taylor R., Smith B.N., Snowden V., Blanc E., Vance C., Topp S., Wong C.H. (2017). Non-nuclear Pool of Splicing Factor SFPQ Regulates Axonal Transcripts Required for Normal Motor Development. Neuron.

[12] Passon D.M., Lee M., Rackham O., Stanley W.A., Sadowska A., Filipovska A., Fox A.H., Bond C.S. (2012). Structure of the heterodimer of human NONO and paraspeckle protein component 1 and analysis of its role in subnuclear body formation. Proc. Natl. Acad. Sci. USA.

[13] Huang J., Casas Garcia G.P., Perugini M.A., Fox A.H., Bond C.S., Lee M. (2018). Crystal structure of a SFPQ/PSPC1 heterodimer provides insights into preferential heterodimerization of human DBHS family proteins. J. Biol. Chem..

[14] Lee M., Passon D.M., Hennig S., Fox A.H., Bond C.S. (2011). Construct optimization for studying protein complexes: Obtaining diffraction-quality crystals of the pseudosymmetric PSPC1-NONO heterodimer. Acta Crystallogr. D Biol. Crystallogr..

[15] Kuwahara S., Ikei A., Taguchi Y., Tabuchi Y., Fujimoto N., Obinata M., Uesugi S., Kurihara Y. (2006). PSPC1, NONO, and SFPQ are expressed in mouse Sertoli cells and may function as coregulators of androgen receptor-mediated transcription. Biol. Reprod..

[16] Shav-Tal Y., Zipori D. (2002). PSF and p54(nrb)/NonO--multi-functional nuclear proteins. FEBS Lett..

[17] Yarosh C.A., Iacona J.R., Lutz C.S., Lynch K.W. (2015). PSF: Nuclear busy-body or nuclear facilitator?. Wiley Interdiscip. Rev. RNA.

[18] Fox A.H., Lam Y.W., Leung A.K., Lyon C.E., Andersen J., Mann M., Lamond A.I. (2002). Paraspeckles: A novel nuclear domain. Curr. Biol..

[19] Sasaki Y.T., Ideue T., Sano M., Mituyama T., Hirose T. (2009). MENepsilon/beta noncoding RNAs are essential for structural integrity of nuclear paraspeckles. Proc. Natl. Acad. Sci. USA.

[20] Naganuma T., Nakagawa S., Tanigawa A., Sasaki Y.F., Goshima N., Hirose T. (2012). Alternative 3′-end processing of long noncoding RNA initiates construction of nuclear paraspeckles. EMBO J..

[21] Banani S.F., Lee H.O., Hyman A.A., Rosen M.K. (2017). Biomolecular condensates: Organizers of cellular biochemistry. Nat. Rev. Mol. Cell Biol..

[22] Yamazaki T., Nakagawa S., Hirose T. (2019). Architectural RNAs for Membraneless Nuclear Body Formation. Cold Spring Harb. Symp. Quant. Biol..

[23] Fox A.H., Nakagawa S., Hirose T., Bond C.S. (2018). Paraspeckles: Where Long Noncoding RNA Meets Phase Separation. Trends Biochem. Sci..

[24] Marko M., Leichter M., Patrinou-Georgoula M., Guialis A. (2010). hnRNP M interacts with PSF and p54(nrb) and co-localizes within defined nuclear structures. Exp. Cell Res..

[25] Geuens T., Bouhy D., Timmerman V. (2016). The hnRNP family: Insights into their role in health and disease. Hum. Genet..

[26] Kim K.K., Kim Y.C., Adelstein R.S., Kawamoto S. (2011). Fox-3 and PSF interact to activate neural cell-specific alternative splicing. Nucleic Acids Res..

[27] Damianov A., Ying Y., Lin C.H., Lee J.A., Tran D., Vashisht A.A., Bahrami-Samani E., Xing Y., Martin K.C., Wohlschlegel J.A. (2016). Rbfox Proteins Regulate Splicing as Part of a Large Multiprotein Complex LASR. Cell.

[28] Rothrock C., Cannon B., Hahm B., Lynch K.W. (2003). A conserved signal-responsive sequence mediates activation-induced alternative splicing of CD45. Mol. Cell.

[29] Melton A.A., Jackson J., Wang J., Lynch K.W. (2007). Combinatorial control of signal-induced exon repression by hnRNP L and PSF. Mol. Cell. Biol..

[30] Heyd F., Lynch K.W. (2010). Phosphorylation-dependent regulation of PSF by GSK3 controls CD45 alternative splicing. Mol. Cell.

[31] Emili A., Shales M., McCracken S., Xie W., Tucker P.W., Kobayashi R., Blencowe B.J., Ingles C.J. (2002). Splicing and transcription-associated proteins PSF and p54nrb/nonO bind to the RNA polymerase II CTD. RNA (N. Y. NY).

[32] Kameoka S., Duque P., Konarska M.M. (2004). p54(nrb) associates with the 5′ splice site within large transcription/splicing complexes. EMBO J..

[33] Rosonina E., Ip J.Y., Calarco J.A., Bakowski M.A., Emili A., McCracken S., Tucker P., Ingles C.J., Blencowe B.J. (2005). Role for PSF in mediating transcriptional activator-dependent stimulation of pre-mRNA processing in vivo. Mol. Cell. Biol..

[34] Hirose T., Virnicchi G., Tanigawa A., Naganuma T., Li R., Kimura H., Yokoi T., Nakagawa S., Benard M., Fox A.H. (2014). NEAT1 long noncoding RNA regulates transcription via protein sequestration within subnuclear bodies. Mol. Biol. Cell.

[35] McKenna N.J., Lanz R.B., O’Malley B.W. (1999). Nuclear receptor coregulators: Cellular and molecular biology. Endocr. Rev..

[36] Dong X., Shylnova O., Challis J.R., Lye S.J. (2005). Identification and characterization of the protein-associated splicing factor as a negative co-regulator of the progesterone receptor. J. Biol. Chem..

[37] Dong X., Sweet J., Challis J.R., Brown T., Lye S.J. (2007). Transcriptional activity of androgen receptor is modulated by two RNA splicing factors, PSF and p54nrb. Mol. Cell. Biol..

[38] Mathur M., Tucker P.W., Samuels H.H. (2001). PSF is a novel corepressor that mediates its effect through Sin3A and the DNA binding domain of nuclear hormone receptors. Mol. Cell. Biol..

[39] Dong L., Zhang X., Fu X., Zhang X., Gao X., Zhu M., Wang X., Yang Z., Jensen O.N., Saarikettu J. (2011). PTB-associated splicing factor (PSF) functions as a repressor of STAT6-mediated Ig epsilon gene transcription by recruitment of HDAC1. J. Biol. Chem..

[40] Bladen C.L., Udayakumar D., Takeda Y., Dynan W.S. (2005). Identification of the polypyrimidine tract binding protein-associated splicing factor.p54(nrb) complex as a candidate DNA double-strand break rejoining factor. J. Biol. Chem..

[41] de Silva H.C., Lin M.Z., Phillips L., Martin J.L., Baxter R.C. (2019). IGFBP-3 interacts with NONO and SFPQ in PARP-dependent DNA damage repair in triple-negative breast cancer. Cell. Mol. Life Sci..

[42] Salton M., Lerenthal Y., Wang S.Y., Chen D.J., Shiloh Y. (2010). Involvement of Matrin 3 and SFPQ/NONO in the DNA damage response. Cell Cycle.

[43] Shi L., Sun J., Kinomura A., Fukuto A., Horikoshi Y., Tashiro S. (2019). Matrin3 promotes homologous recombinational repair by regulation of RAD51. J. Biochem..

[44] Jaafar L., Li Z., Li S., Dynan W.S. (2017). SFPQ*NONO and XLF function separately and together to promote DNA double-strand break repair via canonical nonhomologous end joining. Nucleic Acids Res..

[45] Morozumi Y., Takizawa Y., Takaku M., Kurumizaka H. (2009). Human PSF binds to RAD51 and modulates its homologous-pairing and strand-exchange activities. Nucleic Acids Res..

[46] Petti E., Buemi V., Zappone A., Schillaci O., Broccia P.V., Dinami R., Matteoni S., Benetti R., Schoeftner S. (2019). SFPQ and NONO suppress RNA:DNA-hybrid-related telomere instability. Nat. Commun..

[47] Brookes E., Riccio A. (2019). Location, location, location: Nuclear structure regulates gene expression in neurons. Curr. Opin. Neurobiol..

[48] Lee T.I., Young R.A. (2013). Transcriptional regulation and its misregulation in disease. Cell.

[49] Gabel H.W., Kinde B., Stroud H., Gilbert C.S., Harmin D.A., Kastan N.R., Hemberg M., Ebert D.H., Greenberg M.E. (2015). Disruption of DNA-methylation-dependent long gene repression in Rett syndrome. Nature.

[50] Takeuchi A., Iida K., Tsubota T., Hosokawa M., Denawa M., Brown J.B., Ninomiya K., Ito M., Kimura H., Abe T. (2018). Loss of Sfpq Causes Long-Gene Transcriptopathy in the Brain. Cell Rep..

[51] Iida K., Hagiwara M., Takeuchi A. (2020). Multilateral Bioinformatics Analyses Reveal the Function-Oriented Target Specificities and Recognition of the RNA-Binding Protein SFPQ. iScience.

[52] Wang G., Yang H., Yan S., Wang C.E., Liu X., Zhao B., Ouyang Z., Yin P., Liu Z., Zhao Y. (2015). Cytoplasmic mislocalization of RNA splicing factors and aberrant neuronal gene splicing in TDP-43 transgenic pig brain. Mol. Neurodegener..

[53] Ishigaki S., Fujioka Y., Okada Y., Riku Y., Udagawa T., Honda D., Yokoi S., Endo K., Ikenaka K., Takagi S. (2017). Altered Tau Isoform Ratio Caused by Loss of FUS and SFPQ Function Leads to FTLD-like Phenotypes. Cell Rep..

[54] Iqbal K., Liu F., Gong C.X. (2016). Tau and neurodegenerative disease: The story so far. Nat. Rev. Neurol..

[55] Lowery L.A., Rubin J., Sive H. (2007). Whitesnake/sfpq is required for cell survival and neuronal development in the zebrafish. Dev. Dyn..

[56] Furukawa M.T., Sakamoto H., Inoue K. (2015). Interaction and colocalization of HERMES/RBPMS with NonO, PSF, and G3BP1 in neuronal cytoplasmic RNP granules in mouse retinal line cells. Genes Cells.

[57] Kanai Y., Dohmae N., Hirokawa N. (2004). Kinesin transports RNA: Isolation and characterization of an RNA-transporting granule. Neuron.

[58] Cosker K.E., Fenstermacher S.J., Pazyra-Murphy M.F., Elliott H.L., Segal R.A. (2016). The RNA-binding protein SFPQ orchestrates an RNA regulon to promote axon viability. Nat. Neurosci..

[59] Fukuda Y., Pazyra-Murphy M.F., Tasdemir-Yilmaz O.E., Li Y., Rose L., Yeoh Z.C., Vangos N.E., Geffken E.A., Seo H.S., Adelmant G. (2020). Fast Transport of RNA Granules by Direct Interactions with KIF5A/KLC1 Motors Prevents Axon Degeneration. bioRxiv.

[60] Lu J., Shu R., Zhu Y. (2018). Dysregulation and Dislocation of SFPQ Disturbed DNA Organization in Alzheimer’s Disease and Frontotemporal Dementia. J. Alzheimer’s Dis..

[61] Ke Y.D., Dramiga J., Schutz U., Kril J.J., Ittner L.M., Schroder H., Gotz J. (2012). Tau-mediated nuclear depletion and cytoplasmic accumulation of SFPQ in Alzheimer’s and Pick’s disease. PLoS ONE.

[62] Luisier R., Tyzack G.E., Hall C.E., Mitchell J.S., Devine H., Taha D.M., Malik B., Meyer I., Greensmith L., Newcombe J. (2018). Intron retention and nuclear loss of SFPQ are molecular hallmarks of ALS. Nat. Commun..

[63] Younas N., Zafar S., Shafiq M., Noor A., Siegert A., Arora A.S., Galkin A., Zafar A., Schmitz M., Stadelmann C. (2020). SFPQ and Tau: Critical factors contributing to rapid progression of Alzheimer’s disease. Acta Neuropathol..

[64] Ishigaki S., Riku Y., Fujioka Y., Endo K., Iwade N., Kawai K., Ishibashi M., Yokoi S., Katsuno M., Watanabe H. (2020). Aberrant interaction between FUS and SFPQ in neurons in a wide range of FTLD spectrum diseases. Brain.

[65] Heemels M.T. (2016). Neurodegenerative diseases. Nature.

[66] Gitler A.D., Dhillon P., Shorter J. (2017). Neurodegenerative disease: Models, mechanisms, and a new hope. Dis. Model. Mech..

[67] Ross C.A., Poirier M.A. (2004). Protein aggregation and neurodegenerative disease. Nat. Med..

[68] Elbaum-Garfinkle S. (2019). Matter over mind: Liquid phase separation and neurodegeneration. J. Biol. Chem..

[69] Maziuk B., Ballance H.I., Wolozin B. (2017). Dysregulation of RNA Binding Protein Aggregation in Neurodegenerative Disorders. Front. Mol. Neurosci..

[70] Taylor J.P., Brown R.H., Cleveland D.W. (2016). Decoding ALS: From genes to mechanism. Nature.

[71] Leal S.S., Botelho H.M., Gomes C.M. (2012). Metal ions as modulators of protein conformation and misfolding in neurodegeneration. Coord. Chem. Rev..

[72] Frederickson C.J., Koh J.Y., Bush A.I. (2005). The neurobiology of zinc in health and disease. Nat. Rev. Neurosci..

[73] Caragounis A., Price K.A., Soon C.P., Filiz G., Masters C.L., Li Q.X., Crouch P.J., White A.R. (2010). Zinc induces depletion and aggregation of endogenous TDP-43. Free Radic. Biol. Med..

[74] Garnier C., Devred F., Byrne D., Puppo R., Roman A.Y., Malesinski S., Golovin A.V., Lebrun R., Ninkina N.N., Tsvetkov P.O. (2017). Zinc binding to RNA recognition motif of TDP-43 induces the formation of amyloid-like aggregates. Sci. Rep..

[75] Tyzack G.E., Luisier R., Taha D.M., Neeves J., Modic M., Mitchell J.S., Meyer I., Greensmith L., Newcombe J., Ule J. (2019). Widespread FUS mislocalization is a molecular hallmark of amyotrophic lateral sclerosis. Brain.

[76] Wolozin B., Ivanov P. (2019). Stress granules and neurodegeneration. Nat. Rev. Neurosci..

[77] Mitra J., Guerrero E.N., Hegde P.M., Liachko N.F., Wang H., Vasquez V., Gao J., Pandey A., Taylor J.P., Kraemer B.C. (2019). Motor neuron disease-associated loss of nuclear TDP-43 is linked to DNA double-strand break repair defects. Proc. Natl. Acad. Sci. USA.

[78] Afroz T., Hock E.M., Ernst P., Foglieni C., Jambeau M., Gilhespy L.A.B., Laferriere F., Maniecka Z., Pluckthun A., Mittl P. (2017). Functional and dynamic polymerization of the ALS-linked protein TDP-43 antagonizes its pathologic aggregation. Nat. Commun..

